# Re-organising primary health care to respond to the Coronavirus epidemic in Cape Town, South Africa

**DOI:** 10.4102/phcfm.v12i1.2607

**Published:** 2020-11-05

**Authors:** Robert Mash, Charlyn Goliath, Gio Perez

**Affiliations:** 1 Division of Family Medicine and Primary Care, Faculty of Medicine and Health Sciences, Stellenbosch University, Cape Town, South Africa; 2 Metropolitan Health Services, Western Cape Department of Health, Cape Town, South Africa

**Keywords:** primary health care, service delivery, COVID-19, corona virus, SARS-CoV-2

## Abstract

Cape Town is currently one of the hotspots for COVID-19 on the African continent. The Metropolitan Health Services have re-organised their primary health care (PHC) services to tackle the epidemic with a community-orientated primary care perspective. Two key goals have guided the re-organisation, the need to maintain social distancing and reduce risk to people using the services and the need to prepare for an influx of people with COVID-19. Facilities were re-organised to have ‘screening and streaming’ at the entrance and patients were separated into hot and cold streams. Both streams had ‘see and treat’ stations for the rapid treatment of minor ailments. Patients in separate streams were then managed further. If patients with chronic conditions were stable, they were provided with home delivery of medication by community health workers. Community health workers also engaged in community-based screening and testing. Initial evaluation of PHC preparedness was generally good. However, a number of key issues were identified. Additional infrastructure was required in some facilities to keep the streams separate with the onset of winter. Managers had to actively address the anxiety and fears of the primary care workforce. Attention also needed to be given to the prevention and treatment of non-COVID conditions as utilisation of these services decreased. The epidemic exposed intersectoral and intrasectoral fault lines, particularly access to social services at a time when they were most needed. Community screening and testing had to be refocused due to limited laboratory capacity and a lengthening turnaround time.

## Introduction

South Africa has the most cases of COVID-19 of any country in Africa and at the time of writing Cape Town had the most cases of any district in the country.^1,2^ The Metropolitan Health Services (MHS) in Cape Town were re-organising primary health care (PHC) to respond to the challenges of the epidemic.

Cape Town has a population of approximately 4 million who speak English, Afrikaans and Xhosa.^3^ Approximately 50% of the population have not completed high school, unemployment is around 24%, 36% live below the poverty line and at least 12% of households are informal dwellings. The health services tackle high rates of communicable (HIV and TB) as well as non-communicable diseases (diabetes and hypertension).^4^ In addition, there are high rates of injury and trauma, particularly from interpersonal violence. Maternal and child health also remain an important focus of healthcare.

In recent years, the MHS have started to re-organise their PHC service according to the principles of community-orientated primary care (COPC) (Box 1).^5^ Overall there are 43 primary care facilities of which 10 are open 24-h a day. The metropole’s most vulnerable communities are also served by teams of community health workers (CHWs) who are led by professional nurses and employed by local non-profit organisations (NPOs) in contract to the MHS. A team consists of 10–15 CHWs and each CHW is responsible for approximately 250 households.

Box 1 Principles of the Metropolitan Health Services’ community-orientated primary care approach.^5^

1. Delineation of geographic areas and alignment with primary care facilities and CHW teams
2. Creating PHC teams of 10–15 CHWs led by a professional nurse, and supported by either a nurse practitioner or a medical doctor, or both
3. Forming one functional and integrated team across the facility-based and community-based members
4. Partnership between the MHS and NPOs who employ the CHW teams
5. Defining a generalist and comprehensive scope of practice for team members
6. Supporting the teams and COPC approach with a health information system
7. Engaging with communities around health needs, priorities and assets
8. Engaging with stakeholders around health needs, priorities and assets
9. Training the PHC team for COPC
10. Change management and communication throughout the system

*Source*: Metropolitan Health Services, Cape Town

CHW, community health worker; COPC, community-orientated primary care; MHS, Metropolitan Health Services; NPOs, non-profit organisations; PHC, primary health care.

The provincial response to the COVID-19 epidemic divides the continuum of care into strategies focused on suppression and containment in the community, response of the health services platform, management of adverse outcomes, recovery and vigilance.^6^ In this short report, I am focusing on the re-organisation of both community-based and facility-based PHC services.

## Re-organisation of primary care facilities

Two key principles guided the de-escalation of services at primary care facilities. These were the need to enable social distancing in facilities and the need to prepare for the increase in patients with mild to moderate disease that would be managed in primary care.

Facilities established screening stations for COVID-19 at the entrance and separated patients into ‘hot’ and ‘cold’ streams. Patients with symptoms of COVID-19 (recent onset of sore throat, cough, fever or shortness of breath) were streamed to a testing station where minor ailments could also be seen and treated immediately. If they required further assessment and treatment, they were admitted to the facility in a separate area from which they could also be referred to hospital if necessary. Patients in the ‘cold’ stream were kept separate and minor ailments were also seen and treated immediately at the entrance. Those that needed further assessment and treatment were admitted to the emergency room or centre. Primary care facilities did not intend to intubate or ventilate any suspected COVID-19 patients, but could provide oxygen therapy. Likewise, nebulisation of patients was stopped in favour of the use of spacers for inhaled medication to avoid creating infectious aerosols.

Services for people with new or unstable chronic conditions (e.g. HIV, TB, diabetes, hypertension), pregnant women, family planning, mental health, immunisations and growth monitoring were continued. Stable patients with chronic conditions who were receiving pre-packaged medication were switched to home delivery of medication via community health workers.^7^ Where possible, follow up of people with chronic conditions was devolved to the community health workers in the community.

## Re-organisation of community-based services

The activities performed by CHWs were also re-organised. Activities such as routine household registration and assessment of health risks were stopped during this period. Instead CHWs focused their energy on home delivery of medication and over the first month delivered 184 000 parcels to patients with chronic conditions who were therefore protected from exposure to COVID-19 at health facilities and public transport. In addition, they assisted with community screening and testing that focused on households around known cases in vulnerable communities.^8^ In the first month they assisted with screening of 123 251 people which led to 12 079 tests and identification of 458 new cases. Community health workers were also asked to assist more with the follow up of patients with HIV, TB, mental disorders, postnatal care and palliative care needs in the community.

Community health workers continued to offer physical care to those with impairments or those that needed additional help as a result of the lockdown restrictions. They continued to refer acutely ill children and adults, as necessary, to the primary care facilities and to educate their communities on COVID-19.

## Evaluation of primary care facility preparedness

As part of the re-organisation, the MHS adapted a facility readiness checklist from the National Institute for Communicable Diseases (NICD) (Online Appendix 1) to assess whether facilities had been able to adjust their services to respond to COVID-19. The checklist had 43 items that evaluated facility management, screening and streaming, as well as organisation of hot and cold streams. Key inputs to service delivery were also assessed, such as use of infrastructure, supply chain, people management, health intelligence and communication. At the time of writing, assessments suggested a reasonably high level of facility readiness (Table 1).

**TABLE 1:** Primary care facility readiness (*N* = 28).

Focus area	%
Screen and stream (4 items)	97.3
Cold stream (2 items)	94.6
Infrastructure (4 items)	93.7
Health intelligence (2 items)	89.3
Supply chain (6 items)	88.7
People management (6 items)	88.1
Hot stream (8 items)	85.7
Communication (4 items)	85.7
Management (7 items)	75.5

*Source*: Metropolitan Health Services, Cape Town

## Key reflections and learning

Primary care facilities were able to re-organise their infrastructure to accommodate two streams of patients by using outside spaces and temporary shelters such as gazebos. With the onset of winter weather in Cape Town, the ability to separate streams and maintain social distance was compromised. In response, the MHS planned to rapidly build COVID-19 testing centres at facilities that required additional infrastructure by making use of containers and prefabricated buildings.

People management has been a key area with high levels of staff anxiety and fear over becoming infected with COVID-19. Some facilities were forced to close temporarily when staff became infected with workers threatening strike action and refusing to work in certain areas. As a result of their anxiety, the staff did not always use personal protection equipment (PPE) rationally or according to the guidelines. The realisation that people could be infectious when asymptomatic also meant that staff were not safe in the ‘cold’ stream and needed to use appropriate PPE. Most facilities were quick to train their staff on screening, testing and clinical management of COVID-19 with support from local primary care guidelines.^9^

**FIGURE 1:**
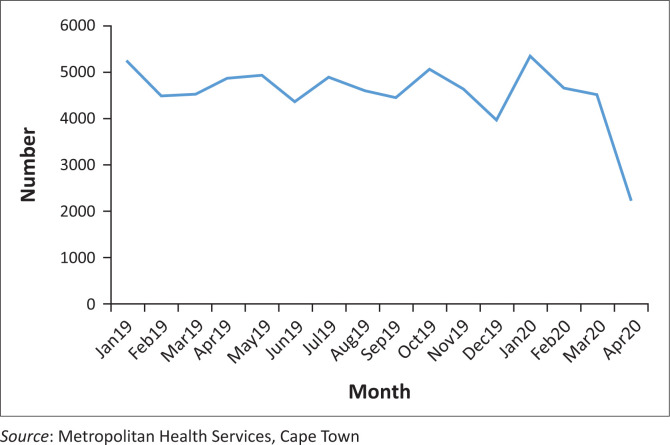
Number of vaccinations for second dose of measles vaccine in Metropolitan Health Services.

As services focused their attention on preparing for and attending to patients with COVID-19, there was a risk that progress made with the control of other diseases would be lost. Initial evidence suggests that this may well be an important issue as, for example, the number of patients tested for HIV, initiated on TB treatment, booking early for antenatal care and obtaining immunisations (Figure 1) all decreased substantially during the lockdown. This was probably a result of messaging to stay at home and fears of attending primary care facilities or using public transport. In addition, CHWs were less focused on identifying and referring people with these conditions to the primary care facilities. The MHS are looking at ways to restore these services during the epidemic.

The foundation that had been previously created for COPC provided a strong basis on which to re-organise services. De-escalating services in primary care facilities and escalating services in the community in a co-ordinated manner was possible because of this groundwork. It is likely that relationships between facility and community-based services and functional integration have improved during this period. One important area of weakness that remains is the ability to work in an intersectoral manner and this is reflected in the low number of referrals by CHWs and the difficulty that facilities have with accessing social services. A further challenge to intrasectoral collaboration was the provision of PHC services by the City of Cape Town in parallel to the MHS. These separate organisations attempted to communicate and collaborate on the re-organisation of services to avoid public confusion and mixed messages.

Another key challenge to community screening and testing has been the increasing turn around time for COVID-19 test results. The backlog of tests has been growing on a daily basis and the turn around time increased to around 10–14 days, making case investigation and contact tracing ineffective. In response to this, CHWs have been asked to only screen households with individuals at high risk of a more severe illness (those > 55 years or with co-morbidity, such as diabetes, hypertension, asthma, poorly controlled HIV or active TB).^10^ Only high-risk individuals and those with more than mild illness will be tested at PHC facilities.

## Conclusion

The MHS in Cape Town have undergone a massive re-organisation of both facility-based and community-based PHC services within a COPC framework in order to respond to the COVID-19 epidemic. The preparedness of PHC facilities appears to generally be at a reasonably high level and as we enter the peak of the epidemic in the next 8–12 weeks, the appropriateness of the preparations will be put to the test. The MHS continue to monitor and revise their plans as the epidemic unfolds.
